# Indoor residential radon exposure and risk of childhood acute myeloid leukaemia

**DOI:** 10.1038/sj.bjc.6690784

**Published:** 1999-11

**Authors:** M Steinbuch, C R Weinberg, J D Buckley, L L Robison, D P Sandler

**Affiliations:** Division of Epidemiology and Clinical Research, University of Minnesota, Minneapolis, MN 55455, USA; Biostatistics Branch, Epidemiology Branch, National Institute of Environmental Health Sciences, Research Triangle Park, NC 27709, USA; Department of Preventive Medicine, Norris Cancer Center MS #44, Los Angeles, CA, 90033-0800, USA

**Keywords:** radon, childhood, acute myeloid leukaemia, epidemiology

## Abstract

Exposure to radon has been identified as a risk factor for lung cancer in uranium miners, but evidence of adverse health effects due to indoor radon exposure is inconsistent. Ecological studies have suggested a correlation between indoor radon levels and leukaemia incidence. We evaluated the risk associated with indoor residential radon exposure within a larger interview-based case–control study of risk factors for childhood acute myeloid leukaemia (AML). A total of 173 cases and 254 controls met the eligibility criteria, and information was collected through telephone interviews with parents and analysis of alpha-track radon detectors placed in the home for a period of 1 year. No association was observed between radon exposure and risk of AML, with adjusted odds ratios of 1.2 (95% confidence interval (CI) 0.7–1.8) for 37–100 Bq m^–3^ and 1.1 (95% CI 0.6–2.0) for > 100 Bq m^–3^ compared with < 37 Bq m^–3^. Although there was an inverse association between radon level and AML risk among children < 2 years at diagnosis, among children ≥2 years, AML risk was increased among those with higher radon exposure. The observed association after age 2 is most likely due to chance. Overall, there was no association between residential radon and risk of childhood AML. © 1999 Cancer Research Campaign


*British Journal of Cancer* (1999) **81**(5), 900–906
© 1999 Cancer Research Campaign
Article no. bjoc.1999.0784

### Indoor residential radon exposure and risk of childhood acute myeloid leukaemia

**M Steinbuch[1], CR Weinberg[2], JD Buckley[4], LL Robison[1] and DP Sandler[3]**

[1]Division of Epidemiology and Clinical Research, University of Minnesota, Minneapolis, MN 55455, USA; [2]Biostatistics Branch and [3]Epidemiology Branch, National Institute of Environmental Health Sciences, Research Triangle Park, NC 27709, USA; [4]Department of Preventive Medicine, Norris Cancer Center MS #44, Los Angeles, CA 90033-0800, USA

**Summary** Exposure to radon has been identified as a risk factor for lung cancer in uranium miners, but evidence of adverse health effects due to indoor radon exposure is inconsistent. Ecological studies have suggested a correlation between indoor radon levels and leukaemia incidence. We evaluated the risk associated with indoor residential radon exposure within a larger interview-based case–control study of risk factors for childhood acute myeloid leukaemia (AML). A total of 173 cases and 254 controls met the eligibility criteria, and information was collected through telephone interviews with parents and analysis of alpha-track radon detectors placed in the home for a period of 1 year. No association was observed between radon exposure and risk of AML, with adjusted odds ratios of 1.2 (95% confidence interval (CI) 0.7–1.8) for 37–100 Bq $m^{-3}$ and 1.1 (95% CI 0.6–2.0) for > 100 Bq $m^{-3}$ compared with < 37 Bq $m^{-3}$. Although there was an inverse association between radon level and AML risk among children < 2 years at diagnosis, among children ≥2 years, AML risk was increased among those with higher radon exposure. The observed association after age 2 is most likely due to chance. Overall, there was no association between residential radon and risk of childhood AML. © 1999 Cancer Research Campaign

**Keywords**: radon; childhood; acute myeloid leukaemia; epidemiology

Radioactive radon is an inert gas that migrates from soil and rock and can accumulate in enclosed areas such as homes and underground mines. The radioactive decay of trace amounts of uranium in the Earth's crust through radium is the source of radon, or more precisely the isotope radon-222. Studies have demonstrated a significant and dose-related excess of lung cancer in radon-exposed miners (National Research Council, 1988; Samet 1989; Lubin et al, 1995). Results from studies of residential radon exposure have been less consistent, but together these studies suggest a modest positive association between indoor radon and lung cancer risk (Lubin and Boice, 1997).

Several recent ecologic studies have found increased rates of leukaemia and other cancers in regions with elevated levels of radon in homes (Lucie, 1989; Alexander et al, 1990; Henshaw et al, 1990), although a small case–control study found no association between childhood cancer and radon exposure (Stjernfeldt et al, 1987). Two of the reports suggested that effects may be more pronounced for childhood leukaemias, specifically myeloid leukaemias (Alexander et al, 1990; Henshaw et al, 1990). Despite the considerable skepticism with which these reports were met, more rigorous study of the potential risks associated with radon was felt to be warranted (Peto, 1990).

Through a collaboration between investigators of the Children's Cancer Group (CCG) and the US National Institute of Environmental Health Sciences (NIEHS), measurement of indoor radon was incorporated into an ongoing national case–control interview study of childhood acute myeloid leukaemia (AML) designed to assess the role of environmental risk exposures in the aetiology of AML. Availability of extensive questionnaire data on potential leukaemia risk factors in conjunction with clinical and laboratory data for a large sample of childhood AML cases provided a good opportunity to study a possible association between indoor radon and childhood leukaemia.

Results from a parallel study of childhood acute lymphoblastic leukaemia (ALL) conducted by the CCG and the US National Cancer Institute have recently been reported (Lubin et al, 1998) and do not support a risk for ALL associated with residential radon exposure. Results regarding AML are reported here.

### MATERIALS AND METHODS

### Case and control selection

In the interview study, cases were identified as individuals diagnosed with AML between 1 January 1989 and 31 March 1993, and registered through member institutions of CCG, a paediatric cooperative clinical trials group conducting therapeutic, biological and epidemiological research. Over 120 institutions in the USA and Canada participate in the CCG.

All newly diagnosed patients with AML or myelodysplastic syndrome (MDS), as defined by the French–American–British (FAB) classification, less than 18 years of age at time of diagnosis, were ascertained and eligibility assessed. To be eligible, the family of the index child must have had a telephone in the household in which they resided at the time the child was diagnosed and resided

*Received 9 September 1998*
*Revised 24 February 1999*
*Accepted 1 March 1999*

*Correspondence to:* M Steinbuch, Children's Cancer Group, PO Box 60012, Arcadia, CA 91066-6012, USA

in the USA or Canada for a minimum of 2 weeks prior to the time of diagnosis; the case's physician must have provided consent to contact the parents; and the biological mother must have been available, English-speaking and willing to be interviewed.

Regional population controls were enrolled for comparison with cases. One control per case was selected for each child with AML classified as M0, M1, M2, M4, or M5 morphology. Two controls per case were selected for cases with MDS or AML of M3, M6, or M7 morphology, to increase statistical power for subgroup analyses. Controls were matched to cases on age (± 25% of age at diagnosis, with a maximum allowable difference of 2 years), race (white, black, other) and geography (area code and exchange). Control selection utilized a random digit dialling (RDD) procedure (Robison and Daigle, 1984). Mothers and fathers of study subjects were interviewed separately by telephone. Interviews included detailed information on a wide range of topics, including a complete medical history, parental occupational history, household exposures, personal habits, parental recreational drug use and postnatal exposures. A focus on specific factors, including pesticides, solvents and petroleum products, maternal marijuana, parental cigarette smoking and alcohol consumption use was included in an effort to confirm previous results (Buckley et al, 1989; Robison et al, 1989; Severson et al, 1993).

Eligibility criteria for participation in the radon measurement component of the study included: (1) the residence of the child at the time of diagnosis (index residence) was occupied at time of interview by one or both parents of the study subject; (2) the entrance to the index residence was no higher than the floor above street level; (3) the parent currently occupying the index residence did not plan to move within the subsequent 6 months; and (4) the child lived in the index residence a minimum of 5 years prior to the time of leukaemia diagnosis (or their entire lifetime for those cases under 5 years of age at diagnosis). This last criterion requiring a minimum time spent in the index residence accounted for most of the 300 cases and 313 controls that were not considered eligible for the radon measurement study. Nineteen cases with Down's syndrome were excluded from the radon measurement study due to their approximate 14-fold higher risk of AML compared to the general population and the potentially distinct aetiology associated with this condition (Robison et al, 1984).

### Radon measurements and exposure assessment

Upon completion of the telephone interview, eligible cases and controls were asked to participate in the radon measurement study. A total of 173 cases and 254 controls fulfilled the eligibility requirements and participated. Each willing participant was mailed general background information about indoor radon, two alpha-track radon detectors (TechOps-Landauer, Glenwood, IL, USA), instructions for the placement of detectors, a toll-free telephone number to call with any questions about the monitors and a brief interview guide, for use during a follow-up telephone interview. A sample of subjects were mailed a third detector for side-by-side placement with another detector for the purpose of quality control. Another quality assurance method involved blind reading of spiked and blank detectors.

Participants were telephoned by an interviewer 2 weeks after detectors and related materials were mailed. Interviewers verified that detectors had been received, answered questions about indoor radon and the radon monitors, and obtained supplemental residential history data. The 5- to 10-min interview enabled interviewers to determine the appropriate locations for monitor placement and ascertain how much time the child usually spent on different levels of the home, outdoors, at school and in other indoor locations.

Participants were instructed to place one detector in the child's bedroom and a second detector in a designated room on the lowest living level on which the child spent a substantial amount of time during the day. In homes where the bedroom and usual daytime room were on the same level, the lowest level of the home where the subject spent the second greatest number of hours was chosen for the second detector. In homes with only one level, one monitor was placed in the child's bedroom and the other was usually placed in the living room or den. Participants were instructed to tack the monitors to the wall above the door or another location away from windows and air returns in the designated rooms, record the date the monitors were unsealed and placed, and return a completed radon detector tracking form with the signed consent form. Monitor placement was verified by comparing rooms listed on the tracking form returned by the respondent with the indicated rooms as instructed by the interviewer.

Six months after monitor placement, a telephone contact was made to confirm monitor status, remind participants that the study was ongoing and reinforce the importance of notifying the study office in the event of problems or loss of monitors and/or plans to move from the index residence. One year after monitor placement, participants were mailed packaging materials and instructions for returning monitors. Study participants requesting results of radon measurements for their home were sent a copy of the results and an Environmental Protection Agency (EPA) brochure on radon.

While radon exposure was measured in pico-Curies per litre (pCi $l^{-1}$), all of the results are reported in Becquerels per cubic metre (Bq $m^{-3}$). The conversion factor is 37 Bq $m^{-3}$ to 1 pCi $l^{-1}$. Remediation of radon is recommended for homes that exceed 148 Bq $m^{-3}$ by the US EPA, and 200 Bq $m^{-3}$ by the British National Radiological Protection Board (NRPB).

Data were collected on the number of waking and sleeping hours spent on different levels of the index home and at other indoor and outdoor locations from birth through the reference/diagnosis date. To better estimate hours spent at home, summary information was collected for two time periods: before and after the child first attended school or day care at least 30 hours per week. We computed a time-weighted average (TWA) radon concentration, simultaneously weighting the radon concentration specific to each level of the home by the average time spent on each level, based on reported activity patterns for the two time periods. Measurements were not made in previous homes or in other indoor or outdoor locations. Time spent away from home was included as a separate variable in statistical models estimating the relative risks associated with residential radon exposure. In 14% of homes no radon measurement was available for a level of the home where the index child spent one or more hours per day. In this situation, a valid measurement for another level was used to estimate the radon concentration on the missing level, based on regression models derived using data from all case and control homes where valid measures were available for both of the relevant levels. These regression relationships were quite linear, with high correlation ($r = 0.81$ for below versus at ground level, $r = 0.97$ for at versus above ground level).

An unmatched analysis was performed since a substantial loss in sample size would result if analyses were restricted to case–control pairs. Risk from radon exposure was evaluated taking into consideration other risk factors that had been identified

© 1999 Cancer Research Campaign

**Table 1** Study participation rates for interview and radon measurement studies

| | Cases | Controls |
|---|---|---|
| | *n* (%) | *n* (%) |
| Eligible participants for interview study | 638 | 771 |
| Maternal interview completed | 530 (83.1) | 612 (79.4) |
| Non-response | 108 (16.9) | 159 (20.6) |
| Physician refusal | 28 (4.4) | – |
| Parental refusal | 53 (8.3) | 142 (18.4) |
| Lost to follow-up | 13 (2.0) | 13 (1.7) |
| Other[a] | 14 (2.2) | 4 (0.5) |
| Matched sets for interview study (at least one control per case) | 517 (81.0) | 610 (79.1) |
| Ineligible subjects for radon measurement study | 300 | 313 |
| Eligible participants in radon measurement study | 217 | 297 |
| Refusal | 13 (6.0) | 11 (3.7) |
| Invalid[b] | 31 (14.3) | 32 (10.8) |
| Radon measurement study participants | 173 (79.7) | 254 (85.5) |

[a]Mother not available, language. [b]Radon detectors not retrieved or placement not confirmed.

in the interview study. The radon-associated relative risks of AML were estimated by calculating odds ratios (OR) as the estimate of relative risk. Adjusted ORs and 95% confidence intervals (CI) were derived using unconditional logistic regression (Breslow and Day, 1980). Final models were adjusted for maternal education, family income, maternal race and age. Taking into account gender and the number of hours per day the index child spent away from home (outdoors or at other indoor locations) did not appreciably alter the results. Tests for linear trend were based on the logistic regression analysis treating the separate categories of radon concentration as ordinal. Because childhood environmental exposures are less likely than pregnancy and parental factors to affect risk for infant leukaemia, we estimated the risk associated with radon separately for children younger than 2 years of age and age 2 and over. Analyses were also conducted for the subset of subjects who lived only in one home (lifetime subset), allowing for assessment of lifetime household radon exposure and minimizing the misclassification of lifetime exposure that results from measuring only the current home.

### RESULTS

Table 1 summarizes the participation rates and reasons for non-response. A total of 173 case and 254 control families participated in the radon measurement study.

Radon measurement error was assessed by comparing the paired readings based on side-by-side detectors placed in 37 (9%) of the study participant homes. The coefficient of variation (CV) was 9%. In addition, there were 50 spiked radon detectors exposed in a controlled laboratory setting (RUST Geotech, Incorporated, Grand Junction, CO, USA) at three separate radon exposures in Bq day$^{-1}$ m$^{-3}$ (364.3, 730.6 and 1088). The mean reported exposure in Bq day$^{-1}$ m$^{-3}$, respectively, were 420, 767 and 1138, resulting in small differences between the target exposures and the mean reported exposures. The overall CV was 13.5% for the spiked radon detectors. Of the 32 blank detectors tested, 19 (59%) were reported as below the detectable threshold of 3 Bq m$^{-3}$, and the average radon level for the remaining 13 detectors was 6 Bq m$^{-3}$. Although they provide some reassurance that dosimetric errors were small, a source of error that these quality assurance measures cannot address is year-to-year variability in the actual radon levels, which can produce a mismatch between the level that was measured and the risk-relevant exposure that was experienced by the study participant.

The frequency and percentages of cases and controls for selected demographic variables are provided in Table 2 for all subjects and for those living their entire life in the measured home. Descriptive statistics for the TWA radon concentration for cases and controls are presented in Table 3. The arithmetic mean of the time-weighted radon concentrations for cases and controls was 49.8 and 56.0 Bq m$^{-3}$, respectively, in the overall population, and 50.9 and 54.0 Bq m$^{-3}$, respectively, in the lifetime subset.

In examination of the aggregate data, no dose–response relationship was observed between risk of AML and postnatal indoor

**Table 2** Frequency distribution for demographic variables

| | Overall (*n* = 427) | | Lifetime subset (*n* = 284) | |
|---|---|---|---|---|
| | Cases | Controls | Cases | Controls |
| **Variables** | *n* (%) | *n* (%) | *n* (%) | *n* (%) |
| Sex | | | | |
| Male | 83 (48.0) | 128 (50.4) | 52 (48.1) | 86 (48.9) |
| Female | 90 (52.0) | 126 (49.6) | 56 (51.9) | 90 (51.1) |
| Maternal education | | | | |
| ≤ High school | 68 (39.3) | 82 (32.3) | 42 (38.9) | 57 (32.4) |
| Post-high school | 63 (36.4) | 88 (34.6) | 37 (34.3) | 62 (35.2) |
| College/post-graduate | 42 (24.3) | 84 (33.1) | 29 (26.9) | 57 (32.4) |
| Maternal race | | | | |
| White | 152 (87.9) | 238 (93.7) | 96 (88.9) | 165 (93.7) |
| Non-white | 21 (12.1) | 16 (6.3) | 12 (11.1) | 11 (6.2) |
| Family income (× 1000) | | | | |
| < $20 | 18 (10.4) | 26 (10.2) | 12 (11.1) | 17 (9.7) |
| $ 20–$40 | 67 (38.7) | 80 (31.5) | 41 (38.0) | 61 (34.7) |
| > $40 | 88 (50.9) | 148 (58.3) | 55 (50.9) | 98 (55.7) |
| Age (years) | | | | |
| < 2 | 37 (21.4) | 59 (23.2) | 37 (34.3) | 59 (33.5) |
| ≥ 2 | 136 (78.6) | 195 (76.8) | 71 (65.7) | 117 (66.5) |

© 1999 Cancer Research Campaign

**Table 3** Mean, median, range and geometric mean of time weighted average radon concentration (Bq $m^{-3}$) for cases and controls in overall and lifetime subset

| | Radon concentration (Bq $m^{-3}$) | | | |
|---|---|---|---|---|
| | Overall | | Lifetime subset | |
| | Cases ($n$ = 173) | Controls ($n$ = 254) | Cases ($n$ = 108) | Controls ($n$ = 176) |
| Arithmetic mean | 49.8 | 56.0 | 50.9 | 54.0 |
| Median | 32.1 | 30.6 | 35.1 | 30.9 |
| Range | 1.7–518.6 | 2.0–558.3 | 1.7–518.6 | 2.0–558.3 |
| Geometric mean | 28.6 | 30.2 | 29.4 | 29.3 |

residential radon exposure, with adjusted ORs of 1.16 (95% confidence interval (CI) 0.7–1.8) and 1.12 (95% CI 0.6–2.0) for 37–100 and > 100 Bq $m^{-3}$ compared with < 37 Bq $m^{-3}$ (Table 4). However, there was a statistically significant age–radon interaction in the overall and in the lifetime subset. Among children < 2 at diagnosis, there was an inverse association between radon level and AML risk (test for trend, $P$ = 0.03), whereas among those ≥ 2 the estimated relative risk was increased among those with higher radon exposure (overall test for trend, $P$ = 0.07; lifetime subset test for trend, $P$ = 0.01) (Table 5). In addition, the adjusted ORs for higher radon exposure increased with increasing age [2–9 years: 1.6 (95% CI 0.7–3.9) and 2.3 (95% CI 0.7–7.3) for 37–100 and > 100 Bq $m^{-3}$ respectively, compared with < 37 Bq $m^{-3}$; 10–17 years: 4.3 (95% CI 1.2–15.4) and 4.6 (95% CI 0.6–39.3) for 37–100 and > 100 Bq $m^{-3}$ respectively, compared with < 37 Bq $m^{-3}$] (data not shown), but the limited sample size precluded finer stratification.

A number of potential confounding variables were examined, including the region where the family lived, the year the home was built, the type of dwelling (single-family versus other), urbanization, opening of windows to ventilate the home, and smoking status of the mother and father of the index child. These factors did not appreciably alter the results in the multiple logistic regression analysis (data not shown). Furthermore, stratification by FAB morphology did not reveal increased risks for specific morphologic subgroups (data not shown). It should be noted, however, that small numbers precluded meaningful analysis by FAB morphology.

### DISCUSSION

Radon has long been recognized to be a lung carcinogen in occupationally exposed underground uranium and hard-rock miners (National Research Council, 1997). Data from studies of miners have been used to estimate that a substantial proportion of lung cancer deaths in the USA could be due to indoor radon (Lubin et al, 1995). Several case–control studies of lung cancer risk associated with residential radon have been published with varying results (Schoenberg et al, 1990; Alavanja et al, 1994;

**Table 4** Risk estimates and corresponding 95% confidence intervals for indoor residential TWA radon concentration and childhood AML

| TWA radon Concentration (Bq $m^{-3}$) | Overall (173 cases and 254 controls) | | | | | | Lifetime subset (108 cases and 176 controls) | | | | | |
|---|---|---|---|---|---|---|---|---|---|---|---|---|
| | No. of cases | No. of controls | Crude OR | 95% CI | OR[a] | 95% CI | No. of cases | No. of controls | Crude OR | 95% CI | OR[a] | 95% CI |
| <37 | 96 | 143 | 1.0 | | 1.0 | | 57 | 98 | 1.0 | | 1.0 | |
| 37–100 | 53 | 76 | 1.04 | 0.7–1.6 | 1.16 | 0.7–1.8 | 37 | 56 | 1.14 | 0.7–1.9 | 1.22 | 0.7–2.1 |
| >100 | 24 | 35 | 1.02 | 0.6–1.8 | 1.12 | 0.6–2.0 | 14 | 22 | 1.09 | 0.5–2.3 | 1.25 | 0.6–2.7 |
| *P*-trend | | | | | 0.58 | | | | | | 0.45 | |

[a]Model adjusted for maternal race, maternal education, family income and age.

**Table 5** Risk estimates and corresponding 95% confidence intervals for indoor residential TWA radon concentration and childhood AML, stratified by age

| Age (years) | TWA radon concentration (Bq $m^{-3}$ | Overall | | | | Lifetime subset | | | |
|---|---|---|---|---|---|---|---|---|---|
| | | No. of cases | No. of controls | OR[a] | 95% CI | No. of cases | No. of controls | OR[a] | 95% CI |
| <2 | | | | | | | | | |
| | <37 | 23 | 22 | 1.0 | | 23 | 22 | 1.0 | |
| | 37–100 | 10 | 25 | 0.32 | 0.1–0.9 | 10 | 25 | 0.32 | 0.1–0.9 |
| | >100 | 4 | 12 | 0.30 | 0.1–1.1 | 4 | 12 | 0.30 | 0.1–1.1 |
| | *P*-trend | | | 0.03 | | | | 0.03 | |
| ≥2 | | | | | | | | | |
| | <37 | 73 | 121 | 1.0 | | 34 | 76 | 1.0 | |
| | 37–100 | 43 | 51 | 1.62 | 1.0–2.7 | 27 | 31 | 2.18 | 1.1–4.4 |
| | >100 | 20 | 23 | 1.59 | 0.8–3.2 | 10 | 10 | 2.79 | 1.0–7.8 |
| | *P*-trend | | | 0.07 | | | | 0.01 | |

[a]Model adjusted for maternal race, maternal education, family income and age.

© 1999 Cancer Research Campaign 

Letourneau et al, 1994; Pershagen et al, 1994). Together, these studies are consistent with the data from miners and suggest a modest positive association between indoor radon and lung cancer risk (Lubin and Boice, 1997). A recent report from the UK (Darby et al, 1998) also supports a link between residential radon exposure and risk of lung cancer.

Evidence for a link between radon and other cancers is more limited. A review of 11 studies of radon exposed miners found no overall increased mortality from all non-lung cancers combined, although increased risks for certain cancers, including leukaemia, were observed (Darby et al, 1995). Several ecological studies suggested the possibility of increased risk for childhood leukaemia (Lucie, 1989; Alexander et al, 1990; Henshaw et al, 1990) and AML (at all ages) (Alexander et al, 1990; Henshaw et al, 1990; Forastiere et al, 1992; Viel, 1993), as well as for other sites (Henshaw, 1990; Eatough and Henshaw, 1993), in regions where average indoor radon concentrations are higher. These reports have been controversial (Bowie, 1990; Mole, 1990; Miller et al, 1993), and a reanalysis of the Henshaw data (Butland et al, 1990) was less suggestive of a radon effect. Other negative studies (Muirhead et al, 1991; Miller et al, 1993) highlight problems of potential confounding when data are based on large units of analysis such as at the regional or country level. A more recent study by Thorne et al (1996) found no difference in overall childhood cancer rates in postcode sectors of England with ‘high’ and ‘low’ radon levels. High radon areas, however, had significantly increased rates of neuroblastoma ($P = 0.02$) and a non-significant increase in childhood AML ($P = 0.11$).

It has been argued that the marrow dose from radon may be greater than predicted from usual dosimetry models and have developed a theoretical model for estimating the dose to bone marrow from radon daughters (Henshaw et al, 1990; Richardson et al, 1991). The biological plausibility of a radon–leukaemia link was further supported by an early report that the frequency of mutations in the HPRT (hypoxanthine phosphoribosyl transferase) gene was associated with radon levels measured in homes (Bridges et al, 1991). A subsequent larger study failed to confirm this link (Cole et al, 1996), although in another study, homozygous mutations of glycophorin A were increased in underground miners with modest radon exposure (Shanahan et al, 1996).

The current study provides little overall evidence of an association between indoor residential radon exposure and risk of childhood AML. However, based on small numbers, there was an apparent interaction between age- and radon-associated risk for AML. Risk was decreased for children younger than 2, but increased for children aged 2 and over. A similar pattern of risk was seen in the Lubin et al (1998) study of radon and ALL, although none of the trends were statistically significant. In that study, odds ratios were below one for children younger than 2, for whom odds ratios decreased with increasing radon concentration. Odds ratios were greater than one for children aged 2–4 and age 10 and older and increased slightly with increasing radon concentration among children 10 and older.

We estimated risks separately for children younger than 2 and age 2 and older because childhood exposures may be less likely than prenatal factors to play an aetiological role in the risk for acute leukaemia diagnosed before age 2. However, the issue of latency between exposure and diagnosis of AML is not straightforward. In contrast to adults, the developing fetus and the growing child may be subject to different pathways for leukaemia induction. The only relevant data on latency is for postnatally exposed children. There was a latency period of several years before observing the peak occurrence of acute leukaemia following high dose radiation exposure among paediatric survivors of Hiroshima and Nagasaki (Ichimaru et al, 1981). Lower dose radiation exposure, such as indoor radon, may require a longer exposure period.

The inverse association observed among children < 2 is unexplained and could be due to chance. On the other hand, AML in infancy may have a distinct aetiology, with possible prenatal origin. Onset during infancy would imply a shorter latency than may be plausible for an environmental factor. While the observed association between radon and AML in children aged 2 and older also could be due to chance, it is also consistent with a possible risk associated with increasing cumulative exposure to radon. The association with radon was most evident among the subset of older children who lived their entire life in the measured residence. For this group, lifetime exposure to radon is least likely to be misclassified, and increasing TWA radon exposure will correlate with age-dependent increasing cumulative exposure.

The mean radon levels observed in this study were substantially lower than both the EPA and NRPB ‘action level’ for radon remediation. These levels are well below those at which substantial increases in risk would be expected, thus raising further questions about the apparent subgroup effects.

When cases are widely distributed geographically, RDD represents a feasible approach for control selection. Not unlike many control selection methods, use of RDD does raise the potential for introduction of selection and subsequent bias since the methodology generally does not allow for detailed characterization of subjects who refuse to provide identifying information or are not reached after repeated attempts. Given this inability to fully characterize non-participants, a systematic selection cannot be ruled out. Moreover, it is unknown if, or how, systematic selection or bias could impact the observed results. As demonstrated in the current study, participating RDD controls are, as a group, of a higher socioeconomic status, requiring adjustment for education and income in the data analysis.

This study improves upon the previous ecological studies and is the largest reported case–control study of residential radon exposure and childhood AML. Other advantages of the study include the direct collection of data from parents of cases and controls and the availability of data on a large number of potential confounding variables. Individual data on radon exposure were obtained via placement of detectors on more than one level of a home for a period of 1 year, and we were able to calculate a measure of radon exposure that took into account the amount of time spent on each level of the home (which may have different levels of radon). We also took into account time spent away from home although we could not directly account for any radon exposure outside of the home. The quality assurance results for side-by-side placement of radon detectors, as well as blank and spiked detectors, provide some reassurance about the quality of the exposure data.

Despite the size of the study, the numbers for subgroup analysis were limited and this precluded estimation of dose–response relationships that may vary with age at diagnosis. Another study limitation relates to not collecting information on possible radon remediation in the index residence which could have been differential between cases and controls. While it would have been desirable to characterize the lifetime radon exposure for all participants, it was not feasible to do so. Therefore, some degree of misclassification of exposure was introduced by the unmeasured years of exposure. For those who lived in more than one home, the TWA

 © 1999 Cancer Research Campaign exposure is less reflective of lifetime exposure than it is for those who had only lived in the measured home. For those with multiple residences, the relevant time-window of exposure may not have been assessed. Furthermore, we did not collect information on maternal residences during pregnancy. Therefore, even among the lifetime subset, prenatal exposures may be misclassified, making it difficult to evaluate the trends for leukaemia in the first 2 years of life.

In summary, this study evaluated the role of indoor residential radon exposure as a possible risk factor for childhood AML. Overall, there was no association between residential radon exposure and AML risk in children. The apparent positive association between radon and risk of AML after age 2 must be interpreted cautiously because of the limited sample size available for subgroup analysis and the lack of consistency with other data on effects of radiation levels as low as those observed in study homes. Nevertheless, further investigation may be warranted.

### ACKNOWLEDGEMENTS

Supported in part by Public Health Service grant CA49450 from the National Cancer Institute, National Institutes of Health, Department of Health and Human Services, and the University of Minnesota Children's Cancer Research Fund. Contributing Children's Cancer Group investigators and grant numbers are provided in the Appendix. The authors wish to thank Drs Matthew P Longnecker, Marilyn Tseng and Jack Brondum for their thoughtful review of this manuscript.

### REFERENCES

Alavanja MCR, Brownson RC, Lubin JH, Berger E, Chang J and Boice JD (1994) Residential radon exposure and lung cancer among nonsmoking women. *J Natl Cancer Inst* **86**: 1829–1837

Alexander FE, McKinney PA and Cartright RA (1990) Radon and leukaemia (letter). *Lancet* **335**: 1336–1337

Bowie SHU (1990) Radon and leukaemia (letter). *Lancet* **335**: 1336

Breslow NE and Day NE (1980) *Statistical Methods in Cancer Research. Vol. 1, The Analysis of Case–control Studies*. IARC Scientific Publication No. 32. International Agency for Research on Cancer: Lyon

Bridges BA, Cole J, Artlett CF, Green MHL, Waugh AP, Beare D, Henshaw DL and Last RD (1991) Possible association between mutant frequency in peripheral lymphocytes and domestic radon concentrations. *Lancet* **337**: 1187–1189

Buckley JD, Robison LL, Swotinsky R, Garabrant DH, LeBeau M, Manchester P, Nesbit ME, Odom L, Peters JM, Woods WG and Hammond GD (1989) Occupational exposures of parents of children with acute nonlymphocytic leukemia: a report from the Childrens Cancer Study Group. *Cancer Res* **49**: 4030–4037

Butland BK, Muirhead CR and Draper CJ (1990) Radon and leukemia (letter). *Lancet* **335**: 1338–1339

Cole J, Green MH, Bridges BA, Waugh AP, Beare DM, Henshaw D, Last R, Liu Y and Cortopassi G (1996) Lack of evidence for an association between the frequency of mutants or translocations in circulating lymphocytes and exposure to radon gas in the home. *Radiat Res* **145**: 61–69

Darby SC, Whitley E. Howe GR, Hutchings SJ, Kusiak RA, Lubin JH, Morrison HI, Tirmarche M, Tomasek L, Radford EP, Roscoe RJ, Samet JM and Yao SX (1995) Radon exposure and cancers other than lung cancer in underground miners: a collaborative analysis of 11 studies. *J Natl Cancer Inst* **87**: 378–384

Darby S, Whitley E, Silcocks P, Thakrar B, Green M, Lomas P, Miles J, Reeves G, Fearn T and Doll R (1998) Risk of lung cancer associated with residential radon exposure in south-west England: a case-control study. *Br J Cancer* **78**: 394–408

Eatough JP and Henshaw DL (1993) Radon and monocytic leukemia in England. *J Epidemiol Community Health* **47**: 506–507

Forastiere F, Quiercia A, Cavariani F, Miceli M, Perucci CA and Axelson O (1992) Cancer risk and radon exposure (letter). *Lancet* **339**: 115

Henshaw DL, Eatough JP and Richardson RB (1990) Radon as a causative factor in induction of myeloid leukemia and other cancers. *Lancet* **335**: 1008–1012

Ichimaru M, Ishimaru T, Mikami M, Yamada Y and Ohkita T (1981) Incidence of leukemia in atomic bomb survivors and controls in a fixed cohort, Hiroshima and Nagasaki, October 1950–December 1978. In: *Radiation Effects Research Foundation Technical Report*. Contract Number: RERF-TR-13-81, Hiroshima (Japan)

Letourneau EG, Krewski D, Choi NW, Goddard MJ, McGregor RG, Zielinski JM and Du J (1994) Case-control study of residential radon and lung cancer in Winnipeg, Manitoba, Canada. *Am J Epidemiol* **140**: 310–322

Lubin JH and Boice JD (1997) Lung cancer risk from residential radon: meta-analysis of eight epidemiologic studies. *J Natl Cancer Inst* **98**: 49–57

Lubin JH, Boice JD, Edling C, Hornung RW, Howe GR, Kunz E, Kusiak RA, Morrison HI, Radford EP, Samet JM, Tirmarche M, Woodward A, Yao SX and Pierce DA (1995) Lung cancer in radon-exposed miners and estimation of risk from indoor exposure. *J Natl Cancer Inst* **87**: 817–827

Lubin JH, Linet MS, Boice JD, Buckley J, Conrath S, Hatch EE, Kleinerman RA, Tarone RE, Wacholder S and Robison LL (1998) A case-control study of childhood acute lymphoblastic leukemia and residential radon exposure. *J Natl Cancer Inst* **90**: 294–300

Lucie NP (1989) Radon and leukaemia (letter). *Lancet* **ii**: 99–100

Miller D, Morrison H, Semenciw R and Mao Y (1993) Leukemia and residential exposure to radon. *Can J Pub Health* **84**: 205–206

Mole RH (1990) Radon and leukaemia (letter). *Lancet* **335**: 1336

Muirhead CR, Butland BK, Green BMR and Draper CJ (1991) Childhood leukemia and natural radiation (letter). *Lancet* **337**: 503–504

National Research Council (1988) *Committee on the Biological Effects of Ionizing Radiation (BEIR IV). Health Risks of Radon and Other Internally Deposited Alpha-emitters*. National Academy Press: Washington, DC

National Research Council (1997) *Committee on the Biological Effects of Ionizing Radiation (BEIR VI). Board on Radiation Effects Research, Commission on Life Sciences, National Research Council, Health Effects of Exposure to Radon*. National Academy Press: Washington, DC

Pershagen P, Akerblom G, Axelson O, Clavensjo B, Damber L and Desai G (1994) Residential radon exposure and lung cancer in Sweden. *N Engl J Med* **330**: 159–164

Peto J (1990) Radon and the risks of cancer. *Nature* **345**: 389–390

Richardson RB, Eatough JP and Henshaw DL (1991) Dose to red bone marrow from natural radon and thoron exposure. *Br J Radiol* **64**: 608–624

Robison LL and Daigle AE (1984) Control selection using random digit dialing for cases of childhood cancer. *Am J Epidemiol* **120**: 164–166

Robison LL, Nesbit ME, Sather HN, Level C, Shahidi N, Kennedy A and Hammond D (1984) Down syndrome and acute leukemia in children: a 10-year retrospective survey from Childrens Cancer Study Group. *J Pediatr* **105**: 235–242

Robison LL, Buckley JD, Daigle AE, Wells R, Benjamin D, Arthur DC and Hammond GD (1989) Maternal drug use and risk of childhood nonlymphoblastic leukemia among offspring: an epidemiologic investigation implicating marijuana (A report from the Childrens Cancer Study Group). *Cancer* **63**: 1904–1911

Samet JM (1989) Radon and lung cancer. *J Natl Cancer Inst* **81**: 745–757

Schoenberg JB, Klotz JB, Wilcox GP, Gil-del-Real MT, Stemhagen A and Mason TJ (1990) Case-control study of residential radon and lung cancer among New Jersey women. *Cancer Res* **50**: 6520–6524

Severson RK, Buckley JD, Woods WG, Benjamin D and Robison LL (1993) Cigarette smoking and alcohol consumption by parents of children with acute myeloid leukemia: an analysis within morphologic subgroup (A report from the Children Cancer Group). *Cancer Epidemiol Biomarkers Prev* **2** 433–439

Shanahan EM, Peterson D, Roxby D, Quintana J, Morely AA and Woodward A (1996) Mutation rates at the glycophorin A and HPRT loci in uranium miners exposed to radon progeny. *Occup Environ Med* **53**: 439–444

Stjernfeldt M, Samuelsson L and Ludvigsson J (1987) Radiation in dwellings and cancer in children. *Pediatr Hematol Oncol* **4**: 55–61

Thorne R, Foreman NK and Mott MG (1996) Radon in Devon and Cornwall and pediatric malignancies. *Eur J Cancer* **32A**: 282–285

Viel JF (1993) Radon exposure and leukemia in adulthood. *Int J Epidemiol* **22**: 627–631

© 1999 Cancer Research Campaign 

### APPENDIX

### Participating principal investigators – Children's Cancer Group

| Institution | Investigators | Grant No. |
|---|---|---|
| Group Operations Center Arcadia, California | W. Archie Bleyer, MD<br>Anita Khayat, PhD<br>Harland Sather, PhD<br>Mark Krailo, PhD<br>Jonathan Buckley, MBBS, PhD<br>Daniel Stram, PhD<br>Richard Sposto, PhD | CA 13539 |
| Univ. of Michigan Medical Center, Ann Arbor, Michigan | Raymond Hutchinson, MD | CA 02971 |
| Univ. of California Medical Center, San Francisco, California | Katherine Matthay, MD | CA 17829 |
| University of Wisconsin Hospital, Madison, Wisconsin | Diane Puccetti, MD | CA 05436 |
| Children's Hospital & Medical Center, Seattle, Washington | J Russell Geyer, MD | CA 10382 |
| Rainbow Babies & Children's Hospital, Cleveland, Ohio | Susan Shurin, MD | CA 20320 |
| Children's National Medical Center, Washington, DC | Gregory Reaman, MD | CA 03888 |
| Children's Hospital of Los Angeles, Los Angeles, California | Paul Gaynon, MD | CA 02649 |
| Children's Hospital of Columbus, Columbus, Ohio | Frederick Ruymann, MD | CA 03750 |
| Columbia Presbyterian College of Physicians & Surgeons, New York, New York | Leonard Wexler, MD | CA 03526 |
| Children's Hospital of Pittsburgh, Pittsburgh, Pennsylvania | A. Kim Ritchey, MD | CA 36015 |
| Vanderbilt Univ. School of Medicine, Nashville, Tennessee | John Lukens, MD | CA 26270 |
| Doernbecher Memorial Hospital for Children, Portland, Oregon | H Stacy Nicholson, MD | CA 26044 |
| University of Minnesota Health Sciences Center, Minneapolis, Minnesota | Joseph Neglia, MD | CA 07306 |
| Children's Hospital of Philadelphia, Philadelphia, Pennsylvania | Beverly Lange, MD | CA 11796 |
| Memorial Sloan-Kettering Cancer Center, New York, New York | Peter Steinherz, MD | CA 42764 |
| James Whitcomb Riley Hospital for Children, Indianapolis, Indiana | Philip Breitfeld, MD | CA 13809 |
| University of Utah Medical Center, Salt Lake City, Utah | William Carroll, MD | CA 10198 |
| Children's Hospital Medical Center, Cincinnati, Ohio | Robert Wells, MD | CA 26126 |
| Harbor/UCLA & Miller Children's Medical Center, Torrance/Long Beach, California | Jerry Finklestein, MD | CA 14560 |
| University of California Medical Center (UCLA), Los Angeles, California | Stephen Feig, MD | CA 27678 |
| University of Iowa Hospitals and Clinics, Iowa City, Iowa | Raymond Tannous, MD | CA 29314 |
| Childrens Hospital of Denver, Denver, Colorado | Lorrie Odom, MD | CA 28851 |
| Mayo Clinic and Foundation, Rochester, Minnesota | Gerald Gilchrist, MD | CA 28882 |
| Izaak Walton Killam Hospital for Children, Halifax, Canada | Dorothy Barnard, MD | – |
| University of North Carolina, Chapel Hill, North Carolina | Stuart Gold, MD | – |
| Children's Mercy Hospital, Kansas City, Missouri | Maxine Hetherington, MD | – |
| Univ. of Nebraska Medical Center, Omaha, Nebraska | Peter Coccia, MD | – |
| Wyler Children's Hospital, Chicago, Illinois | James Nachman, MD | – |
| M.D. Anderson Cancer Center, Houston, Texas | Beverly Raney, MD | – |
| New York Univ. Medical Center, New York, New York | Aaron Rausen, MD | – |
| Childrens Hospital of Orange County, Orange, California | Violet Shen, MD | – |

© 1999 Cancer Research Campaign
